# Revealing the Hidden Relationship by Sparse Modules in Complex Networks with a Large-Scale Analysis

**DOI:** 10.1371/journal.pone.0066020

**Published:** 2013-06-10

**Authors:** Qing-Ju Jiao, Yan Huang, Wei Liu, Xiao-Fan Wang, Xiao-Shuang Chen, Hong-Bin Shen

**Affiliations:** 1 Department of Automation, Shanghai Jiao Tong University, and Key Laboratory of System Control and Information Processing, Ministry of Education of China, Shanghai, China; 2 National Laboratory for Infrared Physics, Shanghai Institute of Technical Physics, Chinese Academy of Science, Shanghai, China; Technical University of Denmark, Denmark

## Abstract

One of the remarkable features of networks is module that can provide useful insights into not only network organizations but also functional behaviors between their components. Comprehensive efforts have been devoted to investigating cohesive modules in the past decade. However, it is still not clear whether there are important structural characteristics of the nodes that do not belong to any cohesive module. In order to answer this question, we performed a large-scale analysis on 25 complex networks with different types and scales using our recently developed BTS (bintree seeking) algorithm, which is able to detect both cohesive and sparse modules in the network. Our results reveal that the sparse modules composed by the cohesively isolated nodes widely co-exist with the cohesive modules. Detailed analysis shows that both types of modules provide better characterization for the division of a network into functional units than merely cohesive modules, because the sparse modules possibly re-organize the nodes in the so-called cohesive modules, which lack obvious modular significance, into meaningful groups. Compared with cohesive modules, the sizes of sparse ones are generally smaller. Sparse modules are also found to have preferences in social and biological networks than others.

## Introduction

Networks that can describe diverse complex systems are successful tools to understand unknown domains in nature [1]. One of the most interesting topics in the area of complex networks is the module structure and its detection. Modular structure detection has received a considerable amount of attention in various fields because of the significant feature that the nodes in the same module have similar attributes. In the literature, great efforts have been devoted to mining cohesive modules of networks in different fields, including social networks [2] such as collaboration networks, technological networks such as the WorldWide Web [3], [4], and biological networks such as protein-protein interaction (PPI) networks [5], metabolic networks [6], and neural networks [7]. A cohesive module indicates its intra-vertices are densely connected, which at the same time are sparsely connected with the vertices in other modules [3], [8], [9]. Detailed analysis on the meanings of the modules can thus be performed on the mining outputs. For example, in protein-protein interaction networks, modules may contain proteins having similar and specific functions within the cell [5]; in metabolic networks, they likely correspond to functional units such as metabolic pathways [10]; in social networks, individuals tend to form modules of similar hobbies, work environment, family, or friends [2].

Although community detection has been widely studied for a long time [11]–[13], early analysis mainly focuses on small networks, which can be accomplished by human power. In recent times, the size of real networks we can measure has grown considerably, reaching millions or even billions of nodes [9]. This calls for a new theoretical framework that uses computer science to deal with such big data for finding relationship among the nodes. Therefore, a large number of computer algorithm-based methods were proposed. One classical method [14] that aims at identification of edges lying between communities appeared in 2002. Another famous approach is the objective function known as modularity [3], [15], which can be used both to discover communities and to measure their strength. By assumption, high values of modularity indicate good partitions. So, several technologies, such as greedy techniques [15], simulated annealing [16], [17], extremal optimization [18], and spectral optimization [1], [19], have been employed to optimize the modularity. Communities in networks often overlap [10], [20], such that nodes can simultaneously belong to several groups. Clique percolation [10] and its derived CFinder [21] have been used for discovering overlapped nodes. In contrast to the above methods, which pay more attention to clustering nodes, link-based community detection [22] can discover both communities and overlapped nodes successfully.

In general, module detection is implemented based on the modular concept that connections of nodes within the same group are denser than connections with the rest of the network. The question is, are there any sparse modules in which the nodes are sparsely connected internally and densely connected with other sparse or cohesive modules possible in complex networks? Some studies on this issue have been reported in biological networks [23]–[25]. In 2010, using an error function, Pinkert et al proposed an alternative approach which does not consider any prior definitions of what actually constitutes a “module” to detect functional modules in PPI networks [23]. They applied their method to the PPI network derived from the Human Protein Reference Database (HPRD) and found some cohesive modules that proved to be functional modules. In addition, the authors also found some significant non-cohesive clusters, which are functionally related and can provide a better description of the PPI network when combined with the cohesive modules. This finding indicated that we need to extend our traditional concept about the functional unit in a complex network by paying more attention to the sparse modules. By overcoming the resolution limit and over-split phenomena of the alternative approach [24], [25], we proposed a BinTree Seeking (BTS) method based on the Edge Density of Module (EDM) and binary tree theory to mine both sparse and cohesive functional modules in biological networks [24]. Experimental results on three real PPI networks demonstrate that functional modules in PPI networks are not dominantly cohesive but can be sparse. Our studies also show that BTS can achieve the goal of mining both the cohesive and sparse modules simultaneously and automatically.

Based on the results obtained in PPI networks, the motivation of this paper is to study whether it is a general principle that sparse modules co-exist with cohesive modules in the same complex network regardless of its type. In order to answer this question, we firstly used BTS method to mine cohesive and sparse modules from 3 real networks with known modular structures. And then these mined cohesive and sparse modules were analyzed in detail by using known brokers in social networks, software classes in computer software networks and functional units, or metabolic pathways in biological networks. We further applied BTS method to 25 different networks including social, computer software, technological and biological networks. As a result, we detected sparse modules in all 25 networks. Although it seems from the results that it is a general rule for a network to have both cohesive and sparse modules, we find the preferences of sparse modules varied on different types of networks. We also illustrate the spatial organization of some of these sparse modules that are found in the real networks, which show that sparse and cohesive modules sometimes are spatially correlated.

## Results

### Comparison to Other Methods

**Figure 1:**
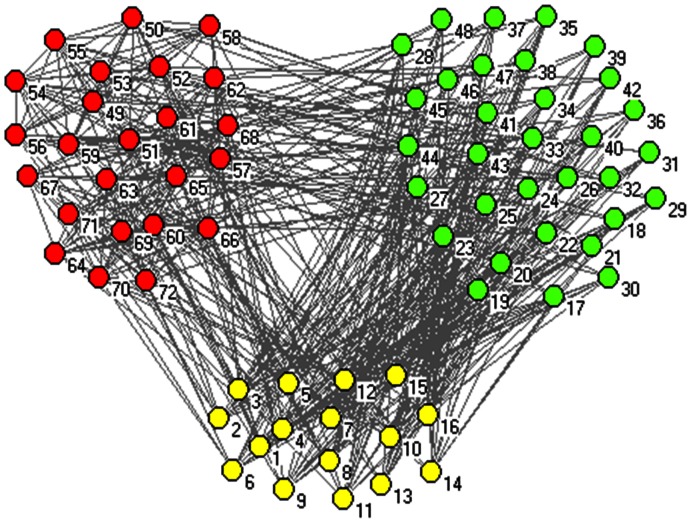
Generated synthetic network of this study.

Although our major aim is not to illustrate the performance of the BTS method, we compared BTS with three other methods on four networks with known modular structure, which are listed in Table 1. The first is a synthetic network that is generated using an algorithm similar to the one used for the SB Benchmark [26] and composed of 72 nodes and 448 edges. The synthetic network comprises three modules of 16, 32 and 24 nodes. Two of these modules are sparse modules and the third one form a cohesive module. The average degrees of the nodes in these communities are fixed to 16, 8, and 16, respectively (See Figure 1). Links are placed according the designed module structure. 12 out of 16 edges in module 1 (light yellow) are linked to module 2 (light green) and the other edges are connected to module 3 (red). Likewise, except for those edges that are connected to module 1, the rest of edges in module 2 are placed between module 2 and 3. For the cohesive module, most edges are connected to intra-module except links that are connected to modules 1 and 2 (The synthetic network data and code are shown in the supplement). The other three real networks are Davis’s southern woman [27], Scottish corpor. interlocks [28], and Jung networks [29] respectively. The Davis network is a well-known bipartite network, which describes the relationship of social collaborations between women in Natchez Mississippi. The other bipartite network is Scottish that supports corporate interlocks in Scotland between 1904 and 1905. The last network is a technological network, where nodes represent software classes and edges correspond to different types of dependencies among them (e.g. inheritance, parameters, variables etc.).

**Table 1:** Performance comparison of four methods on 4 networks.

Network	Nodes	Edges	Number of Real Modules	BTS^a^	NL^b^	Infomod^a^	Pinkert^b^
				NMI (Number of modules)			
Synthesis	72	448	3	0.646(6)	0.423(3)	0.533(4)	0.275(3)
Davis	32	89	4	0.666(2)	0.818(4)	0.669(2)	0.665(4)
Scottish	228	358	9	0.565(5)	0.275(9)	0.122(7)	0.1536(9)
Jung	398	943	38	0.588(35)	0.591(38)	0.537(6)	0.451(38)

a The number of clusters in the network is determined automatically by the algorithms.

b The number of clusters in the network is set according to the number of real modules beforehand.

Different types of modules in these four networks were detected by BTS method. In addition, three methods that deal with the detection of block structures in networks were also applied to these networks. Among them are the mixture model method (NL method) [30] proposed by Newman et al, and the module detection method (Infomod method) [31] by optimizing the function of minimum description length principle. The third compared method is an alternative approach (Pinkert method) [23] that optimizes an error function. Modules detected by the four methods are first compared with known metadata, and then the compared outcomes are measured by normalized mutual information (NMI) [32]. High values of NMI indicate good partitions. The results are presented in Table 1. Note that the parameter of the number of modules is set to the actual number of modules in the NL and Pinkert methods.

From Table 1, BTS performed better than the other methods except on the Davis network, where Infomod performed slightly better and NL performed significantly better than all others due to the number of real modules passed in as input. Overall, the Pinkert method performed poorly.

### The Meaning of Cohesive and Sparse Modules in Various Types of Networks

Although finding the coexistence of cohesive and sparse modules in complex networks is the aim of this study, another more significant task is to reveal their meaning in the complex system. To answer the question whether or not the discovered both sparse and cohesive modules are interesting, we applied BTS to social, computer software and biological networks, respectively, and then analyzed the output modules in detail.

#### Cohesive and sparse modules in social networks

A social network can represent a set of people or groups of people with some relationships between them [33]. These relationships may include friendships between individuals, business partners between companies and intermarriages between families. In contrast to the people in cohesive communities, brokers who trade over gaps in social structure [34], [35] must be credibly connected to actors, but they may not be connected to each other [36]. There are two significant features of brokers. One is that they bridge a gap in social structure, and the other is that they promote information, goods, opportunities, or knowledge flow across that gap [37]. In this section, we applied the BTS method to detect brokers in the well known Newcomb Fraternity network, which contains 17 students living together in a hostel. The development of the network was followed up for 15 weeks except for a holiday break of one week between weeks 9 and 10. In every week, the students were consulted on their friendship preference with the other 16 students [38]. In order to conveniently use BTS method to mine brokers in the Newcomb Fraternity, we transformed it into a network including nodes that represent students and edges that correspond to friendships in the following role: for a student in every week, five links are placed among the student and other five students who are ranked in the top five. Note that an edge that is placed between two students only means that the two students have a strong friendship, does not mean they are unacquainted. According to the BTS consequences (see supporting information at: www.csbio.sjtu.edu.cn/bioinf/SparseNetwork/for more details), in week 8, these students are divided into 5 groups, where groups 4 and 5 are sparse groups including nodes 9 and 17, 13 and 16 respectively. These results are largely consistent with the analysis by Taube [38] that nodes 9, 13 and 17 are coordinators who may mediate contradictions among students. Another example is the network generated by week 13 data. The modular results detected by BTS contain only 1 sparse group, and nodes 13 and 17, who are coordinators, are all in this cluster. Likewise, as a coordinator, node 17 appears in sparse modules in networks generated by weeks 14 and 9 data. We furthermore find that node 17 is observed in many sparse modules corresponding to the 15 week networks, meaning that it plays a significant role in these 17 students.

#### Cohesive and sparse modules in computer software networks

We further used BTS method to analyze a Jung network (in Table 1) in software systems in which the actual structure remains great unknown [29]. In the software network, nodes correspond to software classes and edges represent different types of dependencies among them (e.g., inheritance, parameters, variables etc.) [39]. As a result, we found 14 cohesive and 21 sparse modules respectively. For these modules, we analyzed some modules detected by BTS in detail below. Module 1 with 33 nodes is a cohesive group (See Table 2 and supplement information for detailed results), and 23 of them consist of Jung ‘graph’ class, and the other 10 nodes contain Jung ‘util’ class. As one could anticipate, the nodes in the module are densely connected internally and sparsely connected to the rest of the network. The second cohesive module we analyzed is module 20 that includes 18 nodes, where 13 nodes are divided into the ‘algorithms.scoring’ class and the rest of nodes are clustered into ‘algorithms.shortestpath’, ‘graph’, and ‘io’ classes (more details see Table 2). Three sparse modules are discussed next. Module 5 is a sparse cluster and its size is 20. The surprising result is that all the nodes in module 5 belong to Jung ‘visualization’ class. 9 and 6 nodes in the module 5 participate in ‘visualization.control’ and ‘visualization.renderers’ classes respectively. These results indicate that the nodes in this sparse module detected by BTS share similar attributes. The other two small sparse modules are module 14 and module 15 with 6 and 5 nodes respectively. 4 of 6 nodes in the module 14 contain ‘algorithms.layout3d’ class. In module 15, 4 nodes belong to ‘algorithms.importance’ class. As revealed by the network topology, this module has no intra-group links, but links with other modules, such as sparse modules 14 and 16.

**Table 2:** Analysis of the main modules in Jung network.

Communitynumber	Type	Size	Description
1	cohesive	33	[jung.graph].*(23), .util.*(10).
20	cohesive	18	[jung.algorithms.scoring].*(13), .shortestpath.*(2); [jung.graph].Hypergraph(1); [jung.io].GraphReader(1), .graphml.GraphMLReader2(1).
5	sparse	20	[jung.visualization].*(2),.renderers.*(6),.control.*(9),.annotations.*(2),.transform.LensSupport(1).
14	sparse	6	[jung.algorithms].layout3d.*(4),.flows.EdmonskarpMaxFlow(1),.importance.AbstractRanker(1).
15	sparse	5	[jung.algorithms].importance.*(4),.shortestpath.ShortestPath(1).

* The detailed names of classes are omitted (refer to supporting information at www.csbio.sjtu.edu.cn/bioinf/SparseNetwork/for details).

#### Cohesive and sparse modules in biological networks

In PPI networks, functional subunits or protein complexes generally correspond to modular structures [40]. Recent literatures confirm that sparse clusters that contain few or no edges can form functional units, indicating functional units are not necessarily cohesive modules. In this study, we extend this viewpoint to gene co-expression networks, which are composed of nodes corresponding to genes and edges that represent significant co-expressed relationships between genes [41], [42]. Since genes on the same pathway or have related functions often exhibit similar expression patterns under diverse conditions in DNA microarray experiments [42], therefore, most of works on functional modules or units detection in gene co-expression network pay more attentions on cohesive modules [43]–[45], and rarely on sparse modules. In this section, we first employed BTS method to detect both cohesive and sparse modules in gene co-expression networks, and then discussed the meaning of these modules.

The genes that are used to construct gene co-expression networks are collected from Arabidopsis thaliana metabolic pathway data [46] (www.arabidopsis.org/). By removing repeated genes in the same pathway and pathways with less than 5 genes (in order to avoid many small clusters), we finally obtained 174 pathways that contain 1725 genes. For these 1725 Arabidopsis genes, Arabidopsis gene co-expressed data consisting of 20906 files from the ATTED-II database [47] (http.//atted.jp/) was used to identify their co-expressed relationships. Co-expression was measured using Pearson’s Correlation Coefficients (PCCs). If the PCC of any two genes is higher than 0.6, a link is placed between these two genes. At last, a gene co-expression network with 793 Arabidopsis genes and 10184 edges was constructed (see supporting information).

Using BTS, we detected 14 cohesive and 21 sparse modules from the network. To demonstrate their importance, we compared them with Arabidopsis metabolic pathway data based on the hypothesis that the genes belonging to the same pathway are highly co-expressed [48], [49]. In addition, the functions of these modules can also be measured by BiNGO [50], which is used to assess a set of genes with Gene Ontology (GO) annotations [51]. The cohesive group of Module 5 has 64 nodes, 22 of which participate in the adenosyl-L-methionine cycle. For the rest of genes in this group, 11 nodes and 12 nodes belong to the metabolic pathways of zeatin biosynthesis and galactose degradation respectively. From these results, we can clearly see that most of the genes in Module 5 actively participate in biosynthesis and degradation of adenosyl-L-methionine. Likewise, using BiNGO, we further find that most genes in this module are involved in acetyl-CoA biosynthesis with a low *P-value* (*P-value* = 5.0703E-12, Biological Process (BP)).

Besides cohesive groups, there are also some sparse modules found by BTS. For instance, Module 7 is a sparse module containing 8 nodes. 4 of these 8 nodes are related to the metabolic pathway of cutin biosynthesis, 2 nodes and 1 node belong to chorismate biosynthesis and zeatin biosynthesis respectively. These genes participate in biosynthesis and glucose catabolic process with a significant *P-value* of 1.8822E-9. Another sparse group, Module 1, has 29 genes. These genes participate in different metabolic pathways, but are significantly enriched in small molecule metabolic process (*P-value* = 4.7619E-10). These results demonstrate again that sparse modules not only form significant functional units or participate in metabolic pathways, but also can reveal important hidden relationships among nodes in the network.

### Sparse Modules Co-exist with Cohesive Ones in Complex Networks

To better understand coexistence of cohesive and sparse modules in various complex networks, we further applied BTS approach to 25 networks with different scales and types (Table 3 shows the details). It is revealed by Table 4 (see supporting information for more details) that sparse modules are prevalent in all 25 networks rather than being isolated to specific networks. These results suggest that both cohesive and sparse modules characterize better functional units or modular structure of a complex network than cohesive modules alone. The nodes’ similar functions in a cohesive module are reflected by the direct links among them, while functions in sparse modules are exhibited by indirect linking and depending on other modules. Therefore, the relationship between different nodes should be evaluated by both types of modules.

**Table 3:** Descriptions of 25 networks studied in this paper.

Network Name	Node	Edge	Ref	Description
Csphd (S)	1384	1703	[54]	PH.D. students to their advisors network
Erdos (S)	492	1417	[55]	Erdos collaboration network
Football (S)	115	615	[14]	Network of American football games between Division IA colleges
Lsle_of_Man (S)	675	2007	[54]	The British lsle of Man family of history
Jazz (S)	198	2742	[18]	Jazz musicians network
Science (S)	1589	2742	[56]	A coauthorship network of scientists
Collaboration (S)	5242	14490	[57]	Scientific collaboration network
Roget (S)	1022	5075	[58]	Roget’s thesaurus of English words and Phrases
Geom (S)	7343	11898	[54]	Collaboration network in computational geometry
Java (C)	1538	7817	[55]	Java dependency network
A00 (C)	352	384	[55]	A software project of classes and relationships
A96 (C)	1096	1677	[55]	Finite automaton network
C98 (C)	112	168	[54]	Theorethical graph network
Jung(C)	398	943	[29]	Jung 2.0.1 framework network
E-mail (T)	1133	5451	[59]	Network of E-mail interchanges
Odlis (T)	2909	16380	[60]	Online dictionary of library and information science network
SmallW (T)	396	994	[61]	Citation network produced by HisCite software
Polbook (T)	105	441		Network of books sold by online bookseller
Power (T)	4941	6594	[62]	Power grid network
Usair (T)	332	2126	[54]	United States air line
Yeast PIN (B)	2361	6646	[63]	Protein interaction network in budding yeast
KPI (B)	887	1844	[64]	Protein kinase and phosphatase interaction network
DIP yeast (B)	2147	4275	[65]	Protein interaction network in yeas
BIND human (B)	3724	8748	[66]	Protein interaction network in human
Gene co-expression(B)	793	10184	Gene co-expression network in Arabidopsis	

(S), (C), (T), and (B) indicate social network, computer software network, technological network and biological network, respectively.

**Table 4:** List of modules detected by BTS method in 25 complex networks.

Network name	Cohesive modules	Sparse modules	Total modules
Csphd	5	13	18
Erdos	8	16	24
Football	15	6	21
Isle_of_Man	1	9	10
Jazz	5	12	17
Science	7	17	24
Collaboration	6	19	25
Roget	16	10	26
Geom	4	16	20
Java	9	19	28
A00	8	6	14
A96	7	17	24
C98	3	7	10
Jung	14	21	35
E-mail	7	17	24
Odlis	6	16	22
SmallW	3	2	5
Polbook	7	8	15
Power	5	19	24
Usair	5	9	14
Yeast PIN	5	9	14
KPI	8	21	29
DIP yeast	26	33	59
BIND human	26	39	65
Gene co-expressed	14	21	35

To rationalize the existence of sparse modules, we compared the outputs from BTS and those from the state-of-the-art cohesive-specific module detection approach of Newman-fast algorithm [15] because we do not know the modular structure of these networks. From ref. [52], we know that cohesive modules with less than 10 nodes possibly lack obvious modular significance. However, to provide a full network-based view of the modules, both cohesive modules detected by Newman-fast algorithm of larger and smaller than 10 nodes were considered. For our statistics, these modules are defined as large and small modules respectively. We then can analyze the relationship between the outputs from BTS and Newman method to reveal the inherent mechanism of the sparse module.

**Figure 2:**
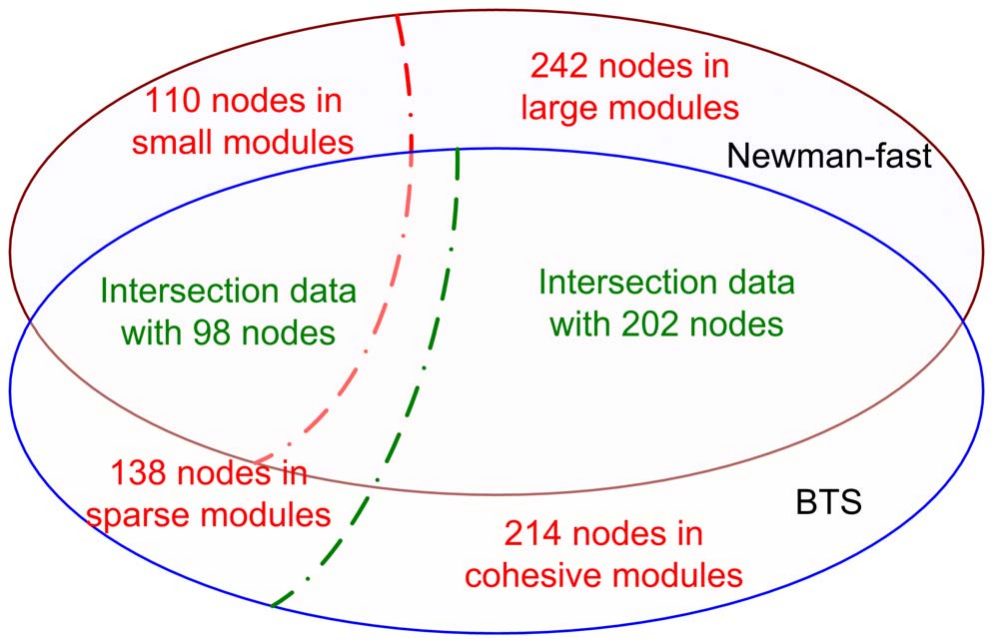
Distributions of nodes in A00 network mined by BTS method and Newman-fast algorithm.

We first compared the results on the A00 network that has 352 nodes and 384 edges. Figure 2 shows the nodes distributions in different types of modules identified by the two methods. In Newman-fast, all the detected modules are cohesive modules; While BTS outputs, both cohesive and sparse modules. As shown in Figure 2, the total number of nodes in the small modules from the Newman-fast approach is 110 and the remaining 242 nodes are located in the large modules. At the same time, we found 138 and 214 nodes in the sparse and cohesive modules respectively from the BTS algorithm. By systematically comparing these outputs, we find that there is an intersection of 98 nodes between the small modules and the sparse modules, and an intersection of 202 nodes between the large modules from Newman-fast approach and the cohesive modules from BTS algorithm. It is also worth mentioning that although there are a large number of overlapped nodes between the small modules from Newman-fast method and the sparse modules from BTS, their node component organizations are quite different. The reason for the differences is that the small modules are subjective to the cohesive definitions in the Newman-fast method, while in the BTS, sparse modules are totally diverse from the cohesive definition [24].

These findings indicate the following: (1) the core set of nodes in the cohesive modules can be well identified by both the cohesive-specific Newman-fast algorithm and the BTS method that can mine both dense and sparse modules; (2) although the modules with less than 10 nodes from the Newman-fast approach possibly lack obvious cohesive modular significance [52], these nodes can be potentially re-organized into sparse modules of important functional units, which should be investigated in a different way from the traditional cohesive-specific approach.

As a second example, let’s analyze modular structures of science network, which describes the cooperative relationship among scientists working on network theory and experiment. This network includes 1461 nodes and 2742 edges (128 isolated nodes are not considered). As a result, 24 modules and 275 modules were mined by BTS method and Newman-fast algorithm respectively. According to BTS, 1140 nodes are divided into sparse modules, and 321 nodes belong to cohesive modules. In the Newman-fast algorithm, 887 nodes belong to small cohesive modules, and the remaining 574 nodes belong to large cohesive modules. By comparing these results, we got a considerable intersection composed of 836 nodes between the 887 nodes of the small modules from Newman-fast algorithm and 1140 nodes in the sparse modules from BTS approach. In addition, 270 nodes are overlapped between the 574 nodes in the large modules from Newman-fast algorithm and 321 nodes in the cohesive modules from BTS.

At last, we compared the results on the Roget network, which has 1022 nodes and 5075 edges. 22 modules including 14 small modules and 8 large modules were mined by Newman-fast algorithm. These small modules contain 47 nodes and the other 975 nodes are in large modules. Using BTS method, we got 16 cohesive modules with 719 nodes and 10 sparse modules with 303 nodes. By comparison, we found that 707 nodes are identical between the cohesive modules from the BTS method and the large modules from the Newman-fast approach.

All these results show that sparse modules co-exist with cohesive ones in various networks. Furthermore, these results also imply that both sparse and cohesive modules can describe better functional groups of a complex network than cohesive modules alone. The reason is that sparse structure can reflect the functional relationship for those nodes in the cohesive modules, which lack obvious traditional modular significance.

### Preferences of Sparse Structures

**Figure 3:**
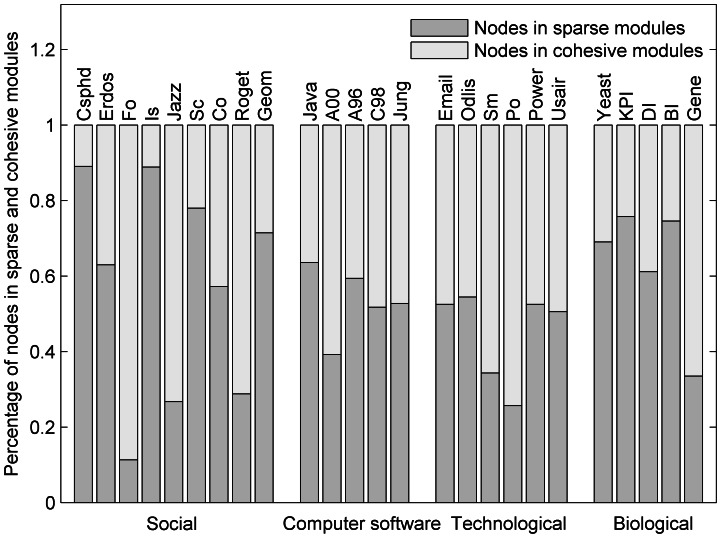
The relative proportions of nodes in different networks from sparse and cohesive modules detected by BTS method.

Which type of networks do sparse modules prefer? It is difficult to answer this question accurately, so we will study this problem in a straightforward way. The relative proportion of nodes in sparse and cohesive modules from BTS is showed in Figure 3, from which we can observe the difference among the different types of networks. In biological networks, high proportions of nodes in sparse modules that may correspond to functional units are found in Figure 3. This implies that biological networks have a higher tendency to possess sparse structure or weakly significant cohesive modules, which is consistent with previous findings [23]–[25].

Significant differences are found in the tested 9 social networks, where 4 of them may show obvious sparse structure (Csphd, Is, Sc, and Geom), and 3 of them have obvious cohesive feature (Fo, Jazz, and Roget). These results reveal that the modular structures in social networks vary very much according to the different relationships represented. For example, in the football network, individuals in a module may communicate with each other frequently or share more related attributes, but exchange rarely between diverse groups. Consequently, those, who share similar attributes, easily form social communities, but few people help information flow across communities. So, few brokers (nodes in sparse modules) are generated in this social network. While, in Csphd and Science networks, in which individuals are engaged in high technology fields, advanced information should be spread across different social communities, and promoting them development fleetly. Therefore, more brokers are needed to perform the task. Another potential reason for this phenomenon in social networks is that current networks are far from complete, and results on a partial network can simply reflect sub-organizations.

Although low proportion of nodes in sparse modules exists in computer software networks shown in Figure 3, it does not mean that sparse modular structures do not exist in them. For instance, we have found some meaningful sparse modules in the Jung and A00 networks as shown above. These results thus seem to imply that sparse modules appear ubiquitous in nature, but have a preference for some types of networks.

### Sizes of Sparse Modules

**Figure 4:**
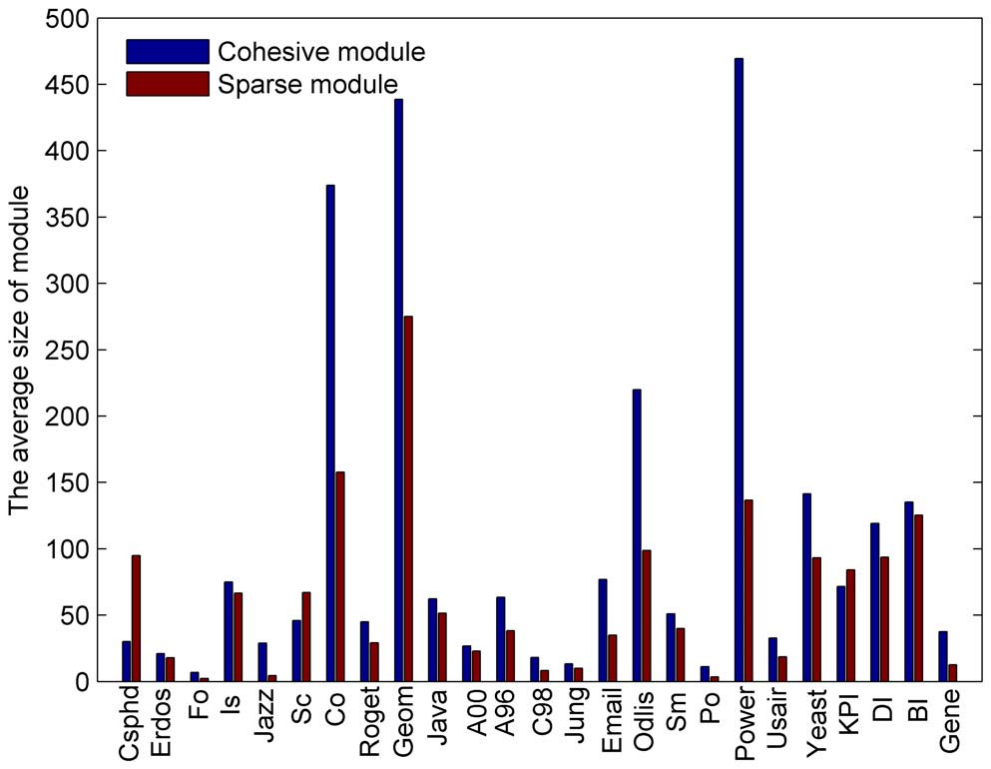
The average sizes of sparse and cohesive modules in various networks.

We further study the size of sparse modules in various networks. Figure 4 compares the average sizes of the mined sparse and cohesive modules in 25 networks by BTS. Generally, sparse modules are smaller than cohesive modules in the same network, although 3 exceptions were observed (Csphd, Sc, and KPI). Here, we give a general explanation for this phenomenon because we do not have enough information on these networks. In social networks, people in sparse modules possibly play the role of brokers or mediator who help information flow across communities or mediate contradictions among people. Thus, these individuals may have low proportion of all people in a special industry. But in some social networks (for example Csphd and Science networks) in which people take part in high technology, the proportion can be larger since this industry needs more people to exchange or share advanced information, to promote their development. Compared to social networks, the ratio of sparse module size to cohesive module size is smaller in software and biological networks, which may be due to sparse modules forming functional units or software packages. However, the fact that the average size of sparse modules is smaller than cohesive modules is still not clear and waiting for further studies.

### Possible Organizing Structures of Sparse Modules

**Figure 5:**
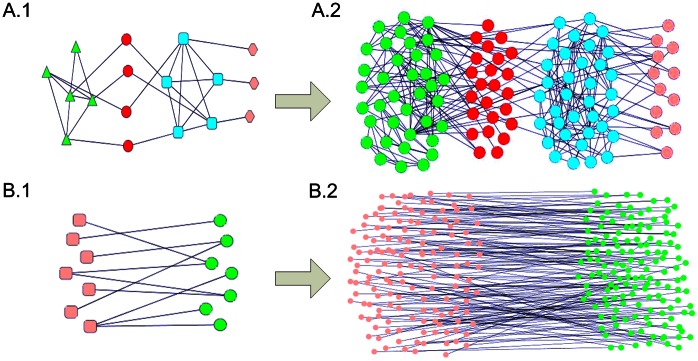
Two possible organizations of sparse modules in the network.

We have shown above that sparse and cohesive modules can simultaneously exist in the same network, but the possible spatial structures of sparse modules are still not clear. Here we report two possible organizing structures for sparse modules in complex networks observed from the results on the 25 networks. Figure 5A.1 illustrates the bridge sparse module that links one or more other cohesive modules, and Figure 5A.2 shows a real example from the Science network. It is also worth pointing out that these bridge modules were also found in the biological networks as reported in refs. [23], [24]. Another common organization is different sparse modules interacting with each other as shown in Figure 5B.1, where Figure 5B.2 illustrates a real example from the Geom network.

### Conclusion and discussion

Complex networks of people, proteins, webpages, or other elements with some pattern of contacts or interactions between them tend to exhibit modular features, which have been studied deeply by sociologists, mathematicians, and biologists. Based on the principle that elements in the same cohesive module have similar attributes, much effort has been devoted to mining cohesive modules and significant progress has been achieved. Recent studies reveal that networks comprise even more sophisticated modules than traditional cohesive modules [30], [39], [53]. For example, sparse modules in PPI networks have been verified to exist, and to have nodes with similar functions [23]–[25]. Using an extended BTS method that can successfully mine both cohesive and sparse clusters in various types of networks, we analyzed the meanings of cohesive and sparse modules detected from three types (social, computer software and biological) of networks. Furthermore, to better show the ubiquitousness of cohesive and sparse modules coexisting in complex networks, the modular structures of 25 different networks were also investigated. Our results suggest that sparse modules commonly exist with cohesive modules, indicating both types of modules should be analyzed simultaneously in order to reveal the functions of the whole network. We also observed some characteristics of the sparse modules and their possible spatial organizations.

Although meaningful results were obtained, great challenges remain. First, in order to further annotate the functions for the mined sparse modules, more information is needed when constructing the networks. This is particularly urgent in social networks since information for verifying the functions of the mined modules is lacking. Second, many functions for measuring cohesive modules have been proposed. But, few functions were developed to assess both cohesive and sparse modules that are simultaneously mined in the same network. Third, algorithms that are suitable for mining both cohesive and sparse modules should be further investigated especially when dealing with large real-world networks (e.g., more than 10000 nodes).

## Datasets and Methods

### Datasets

A total 25 complex networks were studied in this paper, which are summarized in Table 3 (See supplement for the websites of these 25 networks). These networks have following features: (1) different types divided into 4 categories of social, computer software, technological and biological networks, (2) different scales varied from 105 to 7,343 nodes, and from 168 to 16,380 edges, (3) different network topologies reflected by the edge densities. In general, a network can be represented as a graph where a node corresponds to an individual or object and an edge to a special relationship or interaction. For example, Csphd network was constructed to describe the relationship between Ph.D students and their advisors in theoretical computer science, which contains 1384 nodes and 1705 edges.

### Methods

Unlike previous approaches that mainly extract functional units by identifying cohesive modules, we have recently developed an algorithm called BinTree Seeking (BTS) that can also find sparse modules in PPI networks [24]. Members in a sparse module are defined as sparsely connected internally and densely connected with other sparse or cohesive modules at the same time [24]. By using an adjacency matrix to represent a network, BTS detects modules by depicting edges and nodes simultaneously rather than nodes alone and its derivation procedure is based on matrix primary transpositions. When BTS finally converges, it will generate a binary tree, where each leaf represents a state of possible divisions composed of both cohesive and sparse modules. Then, we can use a kind of evaluation function to measure the qualities of the leaf states so that we can pick up the best formulation, e.g. the leaf corresponding to the lowest error function E [23]. The BTS method not only avoids the drawbacks (such as the resolution limit and over-split phenomena [25]) of previous methods but also has some significant merits. One of them is that the number of modules in a network can be automatically determined in this approach. Thus, both sparse and cohesive modules in 25 different networks were mined by BTS algorithm. For more details about the BTS method, the readers are referred to ref. [24], and the main ideas of BTS are given in the appendix. BTS software is available at: http://www.csbio.sjtu.edu.cn/bioinf/BTS/.

To effectively detect cohesive and sparse modules in various networks, three thresholds of (*a*_1_, *a*_2_, *a*_3_) are introduced in BTS method, which play significant roles. *a*_1_ is the lower limit of the link density of cohesive module, *a*_2_ is the upper limit of link density of sparse module, and *a*_3_ is the lower limit of edge density of bridge matrix required to confirm the existence of bridge matrix. In [24], we have discussed the choice of three thresholds in detail on PPI networks. In order to apply BTS method to various types of networks and detect modules effectively, in addition to the classical values of three thresholds (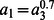, 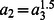, 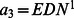, denoted as (0.7, 1.5, 1 ) in a simple form), we also provide here other five groups of three thresholds: (0.5, 1.3, 1), (0.5, 1.7, 1), (0.85, 1.3, 1), (0.8, 1.4, 1) and (0.5, 1.5, 1). Therefore, one can employ BTS method with these thresholds to mine cohesive and sparse modules, and then select a result corresponding to the smallest error function E value or a result that is consistent with known modular structure. Although one can select arbitrary one group of thresholds to detect modules, we suggest that researchers should first consider the classical values of three thresholds because it can generally yield better results. In this study, 11 of 25 networks, synthetic and gene co-expressed networks used the classical thresholds, and 5 networks employed the group of thresholds (0.5, 1.5, 1). The other 3, 2, and 1 networks adopted three thresholds of (0.85, 1.3, 1), (0.8, 1.4, 1), and (0.5, 1.3, 1) respectively (See supporting information for details). All 15 social networks generated by Newcomb Fraternity data used the thresholds of (0.5, 1.7, 1).

**Figure 6:**
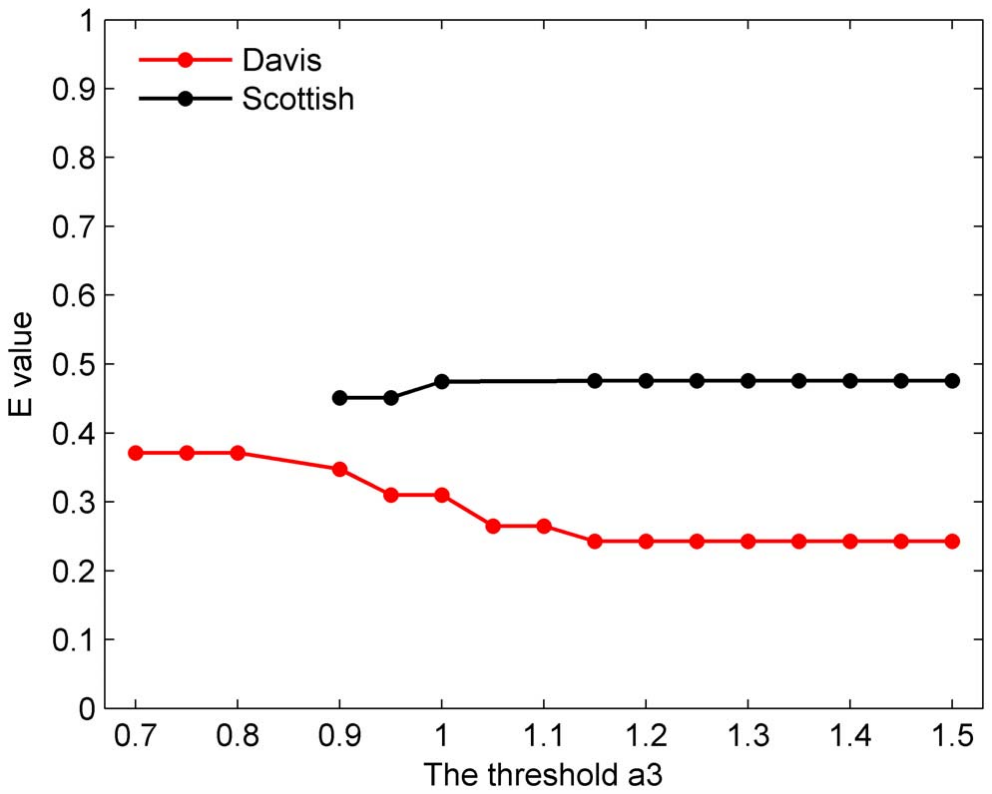
The relationship between a_3_ and E value.

As an improvement over the original BTS method, extended BTS method can also expediently detect modular structure from bipartite networks, such as Divas and Scottish networks. Likewise, detecting bipartite structure also faces the problem of selecting three thresholds. Differing from the problem of three thresholds mentioned above, only one parameter 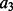 is changed to get better results, and the other two parameters are fixed (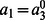, 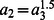). Figure 6 displays the relationship between 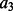 and E values [23]. From Figure 6, we can see that different 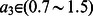 lead to different E values with low fluctuation. So, several values of 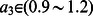 were selected with an interval of 0.05.

## Supporting Information

Text S1 **Brief description of BTS algorithm.**
(DOC)
