# Supplementary material for: Revealing the Hidden Relationship by Sparse Modules in Complex Networks with a Large-Scale Analysis
Source: PLoS One. 2013 Jun 10;8(6):e66020. doi: 10.1371/journal.pone.0066020 (PMC3677904; doi:10.1371/journal.pone.0066020)
Supplement: Text S1 — Brief description of BTS algorithm. (DOC) [file pone.0066020.s001.doc]

**APPENDIX S1: Brief descriptions of BTS algorithm**

In order to distinguish and search cohesive and sparse modules, Edge Density of Module (EDM), Link Density (LD), Edge Density of Bridge Matrix (EDBM), and Edge Density for a Network (EDN) are defined below:

Given a module *r*, its EDM is defined as:

$EDM_{r}=\frac{l_{r}}{n\times(n-1)/2}=\frac{\sum_{i=1}^{n} \sum_{j=1}^{n} r_{ij}}{n^{2}-n}$ (A1)

where $l_{r}$ is the actual number of edges in the module *r*, $n$ is the total number of nodes in *r*. The Link Density (LD) between a node $a$ and a module $r$ is as follows:

$LD=\frac{\sum_{i=1}^{n} e_{ai}}{n}$ (A2)

where $e_{ai}=1$ if there is a link between $a$ and the *i*-th node in *r*, otherwise $e_{ai}=0$.

Edge Density of Bridge Matrix (EDBM) between modules $r_{1}$ and $r_{2}$, and the Edge Density for a Network *R* (EDN) are defined respectively as:

$EDBM=\frac{\frac{1}{2}\sum_{i=1}^{n_{1}} \sum_{j=1}^{n_{2}} bm_{ij}}{n_{1}\times n_{2}}$ (A3)

$EDN=\frac{L_{R}}{N\times(N-1)/2}=\frac{\sum_{i}^{N} \sum_{j}^{N} R_{ij}}{N^{2}-N}$ (A4)

where

$bm_{ij}=\left\{\begin{matrix} 1 i\in r_{1},j\in r_{2}, if vertices i and j are connected, \\ 0 i\in r_{1},j\in r_{2}, if vertices i and j are not connected. \end{matrix}\right.$ (A5)

and $n_{1}$, $n_{2}$ are the number of nodes in modules $r_{1}$ and $r_{2}$ respectively, $L_{R}$ is the actual number of edges in network *R*, $N$ is the total number of nodes in network *R*.

Three matrix blocks are distinguished in BTS, i.e., matrix blocks along the diagonal of the adjacency matrix represent the inner links in the functional modules (both sparse and cohesive modules), and the non-diagonal matrix blocks represent the bridge matrix in the adjacency matrix of a network or the links between different functional modules in a network. To effectively detect the differences of the above three blocks, three thresholds of (*a*_1_, *a*_2_, *a*_3_) are introduced in BTS, which play important roles. *a*_1_ is the lower limit of the link density of cohesive module, *a*_2_ is the upper limit of link density of sparse module, and *a*_3_ is the lower limit of edge density of bridge matrix required to confirm the existence of a bridge matrix.

Starting from an adjacency matrix of a network, BTS will first randomly select a seed node, and then try to build its cohesive modules (left subtree) by adding the nodes whose link density values are larger than *a*_1_, and build its sparse modules (right subtree) by adding the nodes whose link density values are smaller than *a*_2_. As a result, we will obtain a Binary Tree (BinTree) with a root (Adjacency matrix) and two leaves, i.e., left subtree represents the adjacency matrix that includes a cohesive modules and the other represents the adjacency matrix that includes a sparse module. Second, the bridge matrixes of the cohesive modules are built if its edge density of bridge matrix value larger than *a*_3_. Likewise, for the sparse modules obtained by the previous step, its bridge matrixes are also built. Third, remove the nodes whose link density values do not meet the threshold *a*_1_ in cohesive module and regulate the sparse module based on its bridge matrixes. Finally, repeat these steps, until all nodes are processed.

When all the nodes in the network are classified into different modules, a big bintree is built on the whole network and every pathway in the bintree corresponds to a state of decomposing a network into functional units. For all the leaf modules in the bintree, we can then evaluate their qualities using some criteria and find the best outputs. In the current BTS version, the error function E value [1] defined in Eq.(A6) is applied:

$E(\tau,B)=\frac{1}{M}\sum_{i\neq j}^{N} (A_{ij}-B_{\tau_{i}\tau_{j}})(w_{ij}-p_{ij})$ (A6)

where $N$ and *M* are the number of nodes and edges in the network respectively, $\tau$ is the mapping of the $N$ nodes to the $q$ different modules. $A_{ij}$ is the adjacency matrix, $w_{ij}$ represents the weight between nodes $i$ and $j$, if an edge is absent in the network, $w_{ij}$ is zero. $B_{\tau_{i}\tau_{j}}$ is the image graph and $p_{ij}$ is a penalty term.

Refer to [2] for more details about the selections of (*a*_1_, *a*_2_, *a*_3_) and the whole derivation of BTS.

**References**

1. Pinkert S, Schultz J, Reichardt J (2010) Protein interaction networks—more than mere modules. Plos Computational Biology 6: e1000659.

2. Jiao QJ, Zhang YK, Li LN, Shen HB (2011) Bintree seeking: a novel approach to mine both bi-sparse and cohesive modules in protein interaction networks. Plos One 6: e27646.
